# Separate and Shared Neural Basis of Face Memory and Face Perception in Developmental Prosopagnosia

**DOI:** 10.3389/fnbeh.2021.668174

**Published:** 2021-06-25

**Authors:** Xiqin Liu, Xueting Li, Yiying Song, Jia Liu

**Affiliations:** ^1^The Clinical Hospital of Chengdu Brain Science Institute, MOE Key Lab for Neuroinformation, University of Electronic Science and Technology of China, Chengdu, China; ^2^Beijing Key Laboratory of Applied Experimental Psychology, Faculty of Psychology, Beijing Normal University, Beijing, China; ^3^Department of Psychology, Renmin University of China, Beijing, China; ^4^Tsinghua Laboratory of Brain and Intelligence, Department of Psychology, Tsinghua University, Beijing, China

**Keywords:** developmental prosopagnosia, face memory, face perception, core face network, extended face network, individual difference approach

## Abstract

Developmental prosopagnosia (DP), also known as face blindness, is a cognitive disorder with a severe deficit in recognizing faces. However, the heterogeneous nature of DP leads to a longstanding debate on which stages the deficit occurs, face perception (e.g., matching two consecutively presented faces) or face memory (e.g., matching a face to memorized faces). Here, we used the individual difference approach with functional magnetic resonance imaging to explore the neural substrates of DPs’ face perception and face memory that may illuminate DPs’ heterogeneity. Specifically, we measured the behavioral performance of face perception and face memory in a large sample of individuals suffering DP (*N* = 64) and then associated the behavioral performance with their face-selective neural responses in the core face network (CFN) and the extended face network (EFN), respectively. Behaviorally, we found that DP individuals were impaired in both face perception and face memory; however, there was only a weak correlation between the performances of two tasks. Consistent with this observation, the neural correlate of DPs’ performance in face memory task was localized in the bilateral fusiform face area, whereas DPs’ performance in face perception task was correlated with the face selectivity in the right posterior superior temporal sulcus, suggesting that the neural substrates in the CFN for face memory and face perception were separate in DP. In contrast, shared neural substrates of deficits in face perception and face memory tasks were identified in the EFN, including the right precuneus and the right orbitofrontal cortex. In summary, our study provides one of the first empirical evidence that the separate and shared neural substrates of face perception and face memory were identified in the CFN and EFN, respectively, which may help illuminating DP’s heterogeneous nature.

## Introduction

Face recognition plays an important role in our daily life, yet approximately 2–2.9% of the population (Kennerknecht et al., 2006; Bowles et al., 2009) suffers difficulty to recognize faces, which is called developmental prosopagnosia (DP). DP, also known as face blindness, refers to lifelong face recognition deficits since childhood despite normal intelligence, low-level vision, and absence of brain damage (Behrmann and Avidan, 2005; Duchaine and Nakayama, 2006; Susilo and Duchaine, 2013). The deficits seem heterogeneous among DPs (Rivolta et al., 2017; Barton, 2018). While impairment in face memory (e.g., matching a face to memorized faces) characterizes all DPs (Corrow et al., 2016; Dalrymple and Palermo, 2016), some DPs also show deficits in face perception (e.g., discriminating or matching novel faces), which is an early encoding stage before face memory (Bruce and Young, 1986; Behrmann et al., 2005, 2016; Duchaine et al., 2007; Avidan et al., 2011; White et al., 2017), whereas other DPs have intact face perception (Le Grand et al., 2006; Humphreys et al., 2007; McKone et al., 2011; Dalrymple et al., 2014; Ulrich et al., 2017). However, it is unclear how deficits occurring at different stages of face processing lead to the heterogeneous nature of DPs.

One effort to address this issue comes from functional magnetic resonance imaging (fMRI) studies on the neural substrates of DP. According to the most influential neural model (Haxby et al., 2000; Wang et al., 2018; Zhao et al., 2018), brain regions involved in face processing are classified into the core face network (CFN) and the extended face network (EFN). The CFN includes the fusiform face area (FFA) (Kanwisher et al., 1997; McCarthy et al., 1997), the occipital face area (OFA) (Gauthier et al., 2000), and the posterior superior temporal sulcus (pSTS) (Puce et al., 1998), which processes the visual aspects of faces, such as facial features, identity, and expression. The EFN, on the other hand, includes the inferior frontal gyrus, the orbital frontal cortex (OFC), the precuneus, the anterior temporal cortex, and the amygdala, which processes information gleaned from the face, such as biographical and semantic information, emotion, and attractiveness. Previous studies on the neural substrates of DPs mainly focus on the CFN (e.g., Hadjikhani and de Gelder, 2002; Hasson et al., 2003; Bentin et al., 2007; Avidan and Behrmann, 2009; Minnebusch and Daum, 2009; Furl et al., 2011; Rivolta et al., 2012; Avidan et al., 2014), and the EFN is largely ignored (e.g., Wang et al., 2018; Zhao et al., 2018). Methodologically, prior fMRI studies have used univariate or multivariate pattern analysis (MVPA) to investigate face processing measured with different tasks in DP. Early studies have focused on the univariate face selectivity in the CFN (e.g., FFA) of DP individuals and found that DPs showed weakened (Hadjikhani and de Gelder, 2002; Bentin et al., 2007) or normal (Avidan and Behrmann, 2009; Zhang et al., 2015) face selectivity in the FFA. Recent MVPA studies have reported that DPs exhibit abnormal neural activity in both CFN and EFN in face processing tasks (Rivolta et al., 2014; Zhang et al., 2015). Investigations into the brain–behavior correlations offer new insights into the neural basis underlying the behavioral deficits in DP (e.g., Furl et al., 2011; Zhao et al., 2016; Rosenthal et al., 2017). For example, it has been shown that the face selectivity in the FFA is related to face recognition in mixed normal and DP participants (Furl et al., 2011), and recent studies in normal participants also found that the face selectivity or activity pattern in the FFA and OFA could predict face-specific memory (Huang et al., 2014; Ramot et al., 2019), face identification (Tsantani et al., 2021), and holistic face processing (Li et al., 2017). However, these studies did not differentiate neural correlates of face memory and face perception in DP. Here in this study, we measured DPs’ performance in both face perception and face memory tasks and then explored their neural substrates in the CFN and the EFN, respectively.

To do this, we used the individual difference approach in a large sample of DP participants (*N* = 64) to identify the neural correlates of DPs’ face perception and face memory. Behaviorally, we measured DPs’ performance in face perception by matching two consecutively presented novel faces (Song et al., 2015) and in face memory by matching a face with memorized faces (Duchaine and Nakayama, 2004; Zhu et al., 2010). A group of individuals with normal face recognition ability (*n* = 61) was also included for comparison. Neurally, we measured DPs’ face-selective responses when they viewed faces and non-face objects in an fMRI scanner. Finally, we correlated DP’s performance of face perception and face memory with the face-selective responses of the CFN and the EFN to shed light on the heterogeneous nature of DP.

## Materials and Methods

### Participants

Sixty-four DP participants (19–24 years, 28 females) were selected from 9,533 college students at Beijing Normal University and the Chinese Academy of Sciences, China. Based on the diagnosis procedures combining both subjective self-report and objective tests used in previous studies (e.g., Duchaine and Nakayama, 2004, 2005; Garrido et al., 2009; for a review on the guidelines for DP diagnosis, see Dalrymple and Palermo, 2016), we adopted a multiple-stage diagnosis procedure in identifying DPs, including self-report screening, diagnostic interview, diagnostic behavioral test, and validation (for details, see Zhao et al., 2016, 2018). Specifically, in stage 1: screening, self-report questionnaires on face recognition ability in daily life were administered to 9,533 college students, of which 688 questionnaires containing missing values were removed from further analyses. We screened the left 8,845 questionnaires and identified 245 subjects who reported difficulties in face recognition as DP candidates (diagnosis rate: ∼2.7%). In stage 2: diagnostic interview, the 245 DP candidates were individually evaluated with a 1-h semistructured interview on their face recognition ability and related functions, including low-level vision functions related to eyes (e.g., acuity, color vision, astigmatism, strabismus, and presbyopia), object recognition, social behaviors, and neurological diseases (Kennerknecht et al., 2006). Those who reported deficits in low-level vision were excluded from further examination. This interview process resulted in 180 DP candidates (diagnosis rate: ∼73%). This interview process resulted in 180 DP candidates (diagnosis rate: ∼73%). In stage 3: diagnostic behavioral test, an objective test – old/new face memory test (Duchaine and Nakayama, 2004; Wang et al., 2012, 2015) – was conducted to 105 of the 180 potential DPs willing to participate in behavioral tests and resulted in 64 DP participants whose performance was 1 SD below an independent norm of 182 unscreened college students (diagnosis rate: ∼61%). Finally, in stage 4: validation, a paper-based famous-face test was used to confirm whether the 64 DP participants had impairment in face recognition in daily life. Specifically, the test consisted of 30 faces of Chinese celebrities (politicians, movie stars, pop singers, and athletes) with external contours removed, and the participants were instructed to name the celebrities one by one. Another 94 college students from Beijing Normal University [19–28 years, mean = 21.8 (SD = 2.0); 45 females] were tested to construct the norm for the famous-face test. The averaged *z* score of the DP subjects was 3.09, which was significantly below the normal population (*t*_63_ = −24.1, *p* < 0.001, Cohen *d* = −1.7). Thus, these 64 DP participants were identified as participants with DP for further analyses. Therefore, the overall diagnosis rate of DPs in our population was ∼1.2% (2.7, 73, and 61%) or a 2.3 SD cutoff.

In addition, 61 participants (18–29 years, 46 females) with normal face recognition ability participated in the behavioral experiment as normal controls. All participants had normal or corrected-to-normal visual acuity. The normal controls were used only to validate the behavioral deficits of DPs. As the present study focused on the neural basis of behavioral deficits in DPs, the controls were not included in the fMRI data analyses.

Both behavioral and MRI experiments were approved by the Institutional Review Board of Beijing Normal University, Beijing, China. Written informed consent was obtained from each participant before the experiment.

### Behavioral Tests

#### Old/New Face Memory Test

A computer-based old/new face memory test (Duchaine and Nakayama, 2004; Zhu et al., 2010; Song et al., 2015; Yang et al., 2016) was used to measure the face memory ability. The face stimuli consisted of 40 gray-scale pictures of Chinese male faces, all of which had removed the external contours and left a roughly oval shape without hair. The test consisted of two segments: a study segment and a test segment. In the study segment, 10 face stimuli were shown sequentially for 3 s per stimulus with an interstimulus interval (ISI) of 0.5 s. The test segment was immediately after the study segment. In the test segment, the 10 studied (old) faces were shown three times in three blocks, randomly intermixed with 30 new faces. On presentation of each face stimulus, participants were instructed to indicate whether the image had been presented in the study segment. Accuracy was calculated by summing all correct responses and converting to a percentage score to indicate the face memory ability. One participant was excluded because of extreme data (i.e., data that were 3 SDs below the mean) in this test; therefore, the remaining 63 DP participants were included in the further analyses.

#### Face Discrimination Test

A computer-based face discrimination test (Song et al., 2015) was used to measure the facial perception ability. The face stimuli consisted of 40 face images (20 male faces, 20 female faces) with half frontal view and half three-quarter view. The facial stimuli used in the face discrimination test were completely different from those used in the old/new face memory test. There were 40 trials. In each trial, the frontal-view face stimulus was presented at the center of the screen for 0.2 s. After an ISI of 0.5 s, the three-quarter-view face stimulus was presented for another 0.2 s. Participants were instructed to indicate whether the two face stimuli were of the same identity as quickly as possible. Accuracy was calculated by summing all correct responses and converting to a percentage score to indicate the face perception ability.

### fMRI Scanning

The fMRI scan consisted of four-blocked design functional runs, each of which lasted 315 s and contained 16 stimulus blocks (four blocks for each stimulus categories: faces, objects, scenes, and scrambled objects) and five interleaved fixation blocks. In each run, each stimulus category contained 20 images and presented in a 15-s block. Each image was presented for 300 ms at the center of the screen, followed by an ISI of 450 ms. The order of stimulus category blocks in each run was counterbalanced among runs. In the fMRI scan, participants were asked to press a button when two consecutive images were identical.

### MRI Data Acquisition

Magnetic resonance imaging (MRI) data were acquired on a Siemens Trio 3T scanner with a 12-channel phased-array head coil at the Imaging Center for Brain Research, Beijing Normal University. Functional images were collected using a gradient-echo single-slot echo-planar imaging sequence [25 slices, repetition time (TR) = 1.5 s, echo time (TE) = 30 ms, voxel size = 3.125 mm × 3.125 mm × 4.8 mm, field of view (FOV) = 64 mm × 64 mm, flip angle = 90°, interslice gap = 0.8 mm]. High-resolution structural T1-weighted magnetization prepared gradient echo sequence (MPRAGE: 176 slices, TR = 2.53 s, TE = 3.45 ms, inversion time = 1,100 ms, voxel size = 1 mm × 1 mm × 1 mm, FOV = 200 mm × 200 mm, flip angle = 7°). Anatomical scans were also acquired for registration purposes and for anatomically localizing the functional activations. Participants were instructed to remain still, with their heads fixed with foam pads to minimize head motion.

### Data Analysis

#### Image Preprocessing and Face Selectivity

The functional images were processed with FEAT from the Oxford Center for Functional MRI of the Brain Software Library (FSL^1^, Smith et al., 2004) and in-house Python code. The preprocessing steps included motion correction, grand-mean intensity normalization, spatial smoothing (Gaussian kernel, FWHM = 6 mm), and high-pass temporal filtering (high-pass cutoff = 120 s). Each participant’s functional image was aligned to the anatomical image using FLIRT with a linear affine transformation (Jenkinson and Smith, 2001; Jenkinson et al., 2002) and then was normalized to the MNI152 template. The resampled voxel size of functional data was 2 mm × 2 mm × 2 mm.

For each participant, the first-level analysis was conducted separately on each run. The time course of each voxel was fitted by a general linear model (GLM), with each condition (faces, objects, scenes, and scrambled objects) modeled by a boxcar convolved with a γ hemodynamic response function. The temporal derivatives of the convolved boxcars were modeled to improve the sensitivity of the model, and six motion parameters were added to the GLM as covariates. Statistical contrasts between different pairs of categories were evaluated for each participant. Specifically, in the present study, we used the statistical maps from the contrast of faces versus objects to calculate the face selectivity for each voxel. All runs of each participant were combined in a second-level analysis by averaging *z* scores of responses for the faces versus objects from all runs of each participant.

#### Voxel-Wise Brain–Behavior Correlation Analyses

To determine the separate and shared neural basis of face memory and face perception, two separate voxel-wise brain–behavior correlation analyses were conducted in the whole brain to identify the voxels of which the face selectivity significantly associated with the performance of two tasks. Specifically, we used the GLM implemented in the FLAME (FMRIB’s Local Analysis of Mixed Effects) tool of FSL in a voxel-wise manner to map the neural correlates of the out-of-scanner behavioral performance of face memory and face perception, respectively. Gender and age were modeled as confounding factors and were regressed out. Regions that showed significant correlations were identified using the cluster-based correction implemented in AFNI’s 3dClustSim [AFNI version 18.0.25 in which the bug was reported in Eklund et al. (2016) has been fixed in response to control the inflated false-positive rates]^2^. A threshold of cluster-level *p* < 0.05 and voxel-level *p* < 0.01 (two-sided) was set based on Monte Carlo simulations (Ward, 2000; Cox et al., 2016).

For the analysis in the CFN, we restricted the correction to the clusters of contiguous significant voxels under predefined anatomical masks of the occipital--temporal lobes that encompass the CFN: the posterior fusiform cortex, the occipital fusiform cortex, the posterior superior temporal gyrus, the posterior inferior temporal gyrus, the inferior lateral occipital cortex, and the posterior middle temporal gyrus. All masks were taken from the Harvard--Oxford probabilistic structural atlas in FSL (FMRIB, Oxford, United Kingdom, see text footnote 1) with the threshold at 25%. The EFN was defined from the probabilistic activation map (PAM) for faces (faces vs. objects) provided by the brain activity atlas^3^ (Zhen et al., 2015). The face network was defined by keeping the voxels that showed an activation probability higher than 0.1 in the PAM, and the clusters except bilateral OFA, FFA, and pSTS were defined as the EFN (Wang et al., 2018; Zhao et al., 2018).

For the overlap analysis, a less stringent threshold (voxel-level *p* < 0.05, uncorrected) was used to compute the correlation maps of face memory and face perception in both the CFN and EFN, and only overlapping regions with cluster size of greater than 20 contiguous voxels were reported.

## Results

**FIGURE 1:**
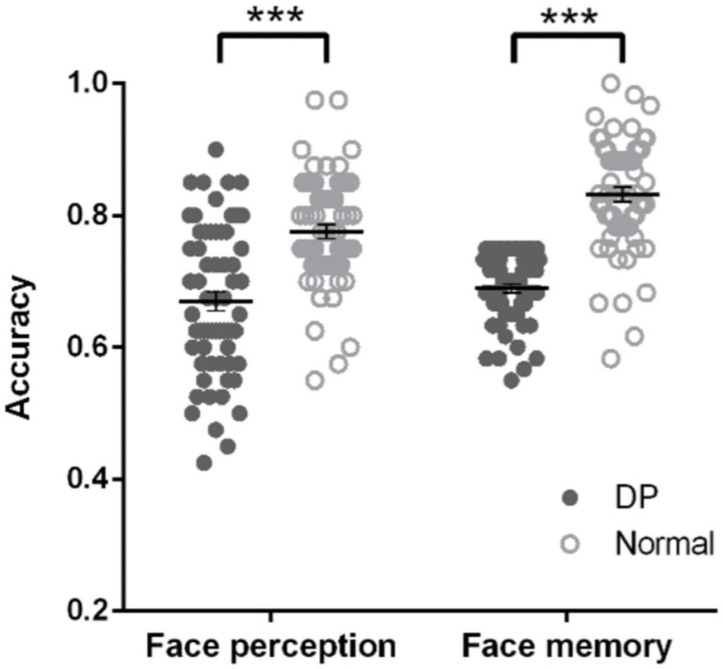
DPs’ behavioral deficits in face memory and face perception. In both face perception task **(left)** and face memory task **(right)**, DPs’ accuracy was significantly lower than that of normal individuals. Error bar shows standard errors of the mean. Each dot shows the individual data point for each participant. ***p < 0.001.

Before identifying the neural correlates of DPs’ face perception and face memory, we first validated the behavioral deficits of DPs by comparing their performance with normal individuals. We found that DPs’ accuracy in both face perception task (*t*_122_ = −5.970, *p* < 0.001, Cohen *d* = −1.07; Figure 1, left) and face memory task (*t*_122_ = −11.107, *p* < 0.001, Cohen *d* = −2.00; Figure 1, right) was significantly lower than that of normal individuals, consistent with previous studies (Bruce and Young, 1986; Behrmann et al., 2005, 2016; Duchaine et al., 2007; Avidan et al., 2011; White et al., 2017). Interestingly, there was a weak but significant correlation in accuracy between these two tasks in DP (Pearson *r* = 0.291, *p* < 0.05), implying that there might be a shared deficit underlying face perception and memory, but only to some extent. Next, we investigated the separate and shared neural correlates underlying DPs’ behavioral deficits.

**FIGURE 2:**
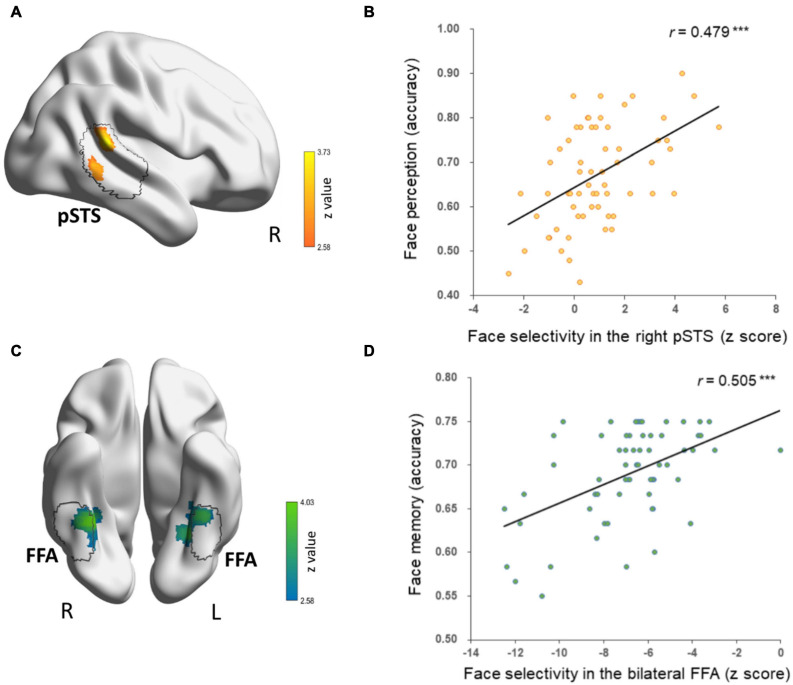
Separate neural correlates of face memory and face perception in DPs’ core face network. **(A)** A cluster in the right pSTS that showed correlation between face selectivity and face perception accuracy (cluster-level *p* < 0.05, voxel-level *p* < 0.01, corrected) is displayed on the surface of MNI152 standard template. The black contour line delineates the right pSTS from the probabilistic activation map (PAM) of face-selective regions in healthy adults. **(B)** Scatter plots between face perception accuracy and *z* scores of face selectivity in the right pSTS are shown for display purpose only. **(C)** The bilateral fusiform clusters that showed correlation between face selectivity and face memory accuracy were located in bilateral FFA from the face-selective PAM in healthy adults, which is outlined in black contour. **(D)** Scatters plots between face memory accuracy and *z* scores of face selectivity in bilateral fusiform clusters are shown for display purpose only.

First, we correlated face-selective neural responses of each voxel across the brain with DPs’ behavioral performance of face perception and face memory, respectively. Only contiguous voxels (*p* < 0.01) that survived cluster-based correction (*p* < 0.05) in the occipital–temporal mask are reported. Two clusters were identified in the CFN. For face perception, we found a cluster in the right pSTS (cluster size: 172, MNI coordinates: 64, −40, 16; Figure 2A) whose face selectivity was positively correlated with face perception in DPs (Figure 2B). That is, the weaker the face selectivity in the right pSTS, the worse performance in face perception. Note that most voxels of this cluster were located within the right pSTS parcels of the PAM for faces at threshold of 10% (Zhen et al., 2015). For face memory, we observed a cluster in the right fusiform gyrus (cluster size: 211, MNI coordinates: 32, −44, −16; Figure 2C) and a cluster in the left fusiform gyrus (cluster size: 205, MNI coordinates: −28, −52, −14; Figure 2C), whose face selectivity was positively related to face memory performance in DPs (Figure 2D). Again, most voxels of these two clusters located within the right and left FFA parcels derived from the PAM for faces at threshold of 10% (Zhen et al., 2015), and then the two clusters were labeled as the left and right FFA. That is, the weaker the face selectivity in the FFA, the worse performance in face memory. Taken together, the neural correlate of face perception was apparently separate from that of face memory in the CFN in DP.

**FIGURE 3:**
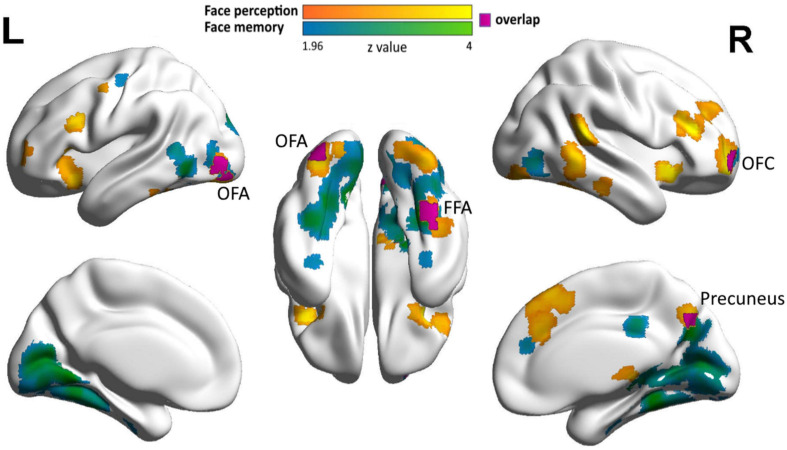
Neural correlates of face memory and face perception in DP in the extended face network. Clusters showing correlations of face selectivity with face memory (cyan), face perception (orange), and their overlap (purple) with a liberal threshold (cluster level: *p* < 0.05, voxel level: *p* < 0.05, uncorrected) are displayed on the surface of MNI152 standard template.

However, one may argue that the dissociative neural correlates might result from the strict threshold (i.e., voxel-level *p* < 0.01, cluster-level *p* < 0.05), under which shared neural substrates in the CFN, if any, failed to survive the statistical analysis. To examine this possibility, we conducted the correlation analyses with a more liberal threshold (voxel-level *p* < 0.05, uncorrected) to explore possible shared neural basis between face perception and face memory in DP. Indeed, with such liberal threshold, three overlapping clusters were identified in the CFN: one in the right FFA (cluster size: 60, MNI coordinates: face memory 34, −44, −16; face perception 38, −48, −20, Figure 3) and the other two in the left OFA (the lateral one, cluster size: 49, MNI coordinates: face memory −38, −82, −4; face perception −38, −82, −2; the ventral one, cluster size: 27, MNI coordinates: face memory −34, −88, −12; face perception −34, −84, −14) (Figure 3). However, careful examination on the correlation between behavioral performance and face selectivity revealed distinct correlation patterns underlying face perception and face memory in the shared clusters. That is, in the shared cluster located in the right FFA, the correlation between face perception accuracy and face responses was positive (Pearson *r* = 0.212, *p* = 0.095), whereas the correlation between face perception accuracy and responses to objects was negative (Pearson *r* = −0.086, *p* = 0.501), and such difference in correlation was significant (Steiger *Z* = 2.18, *p* = 0.029). In contrast, the association between face memory accuracy and face selectivity in this cluster was mainly driven by a significant negative correlation between face memory and neural responses to objects (Pearson *r* = −0.317, *p* = 0.011), whereas the correlation between face memory and the responses to faces was nearly zero (Pearson *r* = 0.080, *p* = 0.534) (Steiger *Z* = −2.94, *p* < 0.005). That is, in the right FFA, the brain–behavior association for face perception was mainly driven by neural responses to faces, whereas the association for face memory was mainly driven by responses to objects.

Similar pattern was also observed in the shared clusters located in the left OFA. That is, the correlation between face perception accuracy and neural responses to faces was positive (Pearson *r* = 0.122, *p* = 0.342, collapsed across the two OFA clusters), whereas the correlation between face perception accuracy and responses to objects was negative (Pearson *r* = −0.132, *p* = 0.303). The difference in correlation was significant (Steiger *Z* = 2.72, *p* = 0.006). In contrast, the correlation between face memory accuracy and responses to objects was strongly negative (Pearson *r* = −0.396, *p* = 0.001), whereas the correlation between face memory and responses to faces was weak (Pearson *r* = −0.171, *p* = 0.180) (Steiger *Z* = 2.53, *p* = 0.011).

Furthermore, we calculated the partial correlation between face selectivity in the overlapping clusters of the right FFA and left OFA and performance in the memory task, with the performance in the perception task regressed out. We found that the correlation between face selectivity and memory accuracy remained significant after controlling the performance in the perception task (the right FFA, *r* = 0.777, *p* = 0.007; the left OFA, *r* = 0.261, *p* = 0.041). These results suggest that the overlapping clusters may be associated with face perception and face memory, respectively, rather than related to some common components shared by the two tasks (e.g., perception component in both tasks).

Taken together, although shared clusters were identified with the liberal threshold, the association between behavioral performance and neural responses likely utilized different mechanisms for face perception and memory.

With such liberal threshold, we also identified two shared clusters in the EFN, one in the right precuneus (cluster size: 29, MNI coordinates: face memory 12, −68, 36; face perception 8, −70, 38) and the other in the right OFC (cluster size: 23, MNI coordinates: face memory 26, 64, −6; face perception 28, 60, −4) (Figure 3). These regions not only showed associations between face selectivity and the accuracy in both face memory and face perception tasks, but also exhibited similar correlational patterns for face memory and face perception. In the right precuneus, neural responses to faces were positively correlated with both face perception accuracy (Pearson *r* = 0.221, *p* = 0.082) and face memory accuracy (Pearson *r* = 0.160, *p* = 0.210), whereas neural responses to objects showed weak and even near-zero correlations with face perception (Pearson *r* = 0.103, *p* = 0.421) and face memory (Pearson *r* = 0.029, *p* = 0.822). Similarly, in the right OFC, neural responses to faces were positively correlated with both face perception accuracy (Pearson *r* = 0.138, *p* = 0.280) and face memory accuracy (Pearson *r* = 0.171, *p* = 0.180), whereas neural responses to objects showed near zero correlations with face perception (Pearson *r* = −0.050, *p* = 0.699) and face memory (Pearson *r* = −0.042, *p* = 0.746). Taken together, the shared neural substrates of face perception and face memory were identified in DPs’ EFN with a liberal threshold.

## Discussion

In the present study, with a large sample of DPs and an individual difference approach, we investigated the relation of deficits in face memory and face perception in the brain. Behaviorally, we found that although DPs showed deficits in both face perception and face memory, there was only a weak correlation between the performances of these two tasks. Neurally, the neural correlate of face memory was dissociable with that of face perception in the CFN. Specifically, the neural correlate of DPs’ face memory was localized in the bilateral FFA, whereas DPs’ face perception was associated with the face selectivity in the right pSTS. Moreover, even with three overlapping clusters in the CFN identified with a liberal threshold, the pattern of the brain–behavior correlation was different between the two tasks. That is, the neural correlate of face memory was separate from that of face perception in DP in the CFN. In contrast, shared neural basis of face memory and face perception was identified in the EFN, including the right precuneus and the right OFC, further confirmed by a similar brain–behavior correlation pattern between the two tasks in these two regions. Together, these findings demonstrated that DPs’ face perception and face memory had both separate and shared neural correlates yet in different face networks, echoing the significant but weak correlation in performing these two face tasks in DP. In short, our finding of distinct relation of face memory and face perception in two face networks may help shedding light on how deficits at different stages of face processing lead to the heterogeneous nature of DPs.

Previous studies have shown that the FFA is a pivotal brain area involved in face recognition, where DPs show abnormal face selectivity (Hadjikhani and de Gelder, 2002; Bentin et al., 2007; Minnebusch and Daum, 2009; Furl et al., 2011; Song et al., 2015). Neuropsychological studies further provide a direct link between the FFA and behavioral performance in face recognition that lesion or stimulation of the FFA can lead to selective impairment in face recognition (Damasio et al., 1982; Sergent and Signoret, 1992; Barton et al., 2002; Parvizi et al., 2012). In line with these studies, our study for the first time demonstrated that the face selectivity in the FFA was also predictive of DPs’ behavioral performance in face memory task. The present finding extended previous studies demonstrating the association between FFA face selectivity and face memory performance in normal participants (Huang et al., 2014; Ramot et al., 2019) and across DP and normal participants (Furl et al., 2011). Notably, although the face selectivity in FFA is related to face memory ability in both DPs and normal participants, the pattern of the brain–behavior correlation is different. For normal people, the association is mainly driven by the correlation between face memory and FFA responses to faces (Huang et al., 2014), whereas for DPs, the association was mainly driven by the FFA responses to objects found in the present study. These results suggest that although the FFA is involved in face memory in both DPs and normal participants, it may mediate face memory in DPs and normal participants through different mechanisms.

Interestingly, the brain–behavior correlation for face perception was not observed in the FFA; instead, the association was found in the right face-selective pSTS, which is an important area in the CFN specialized in processing both face expression and face identity (Winston et al., 2004; Fox et al., 2009; Yang et al., 2016). Consistent with our observation, a neuropsychological study on acquired prosopagnosia has reported a patient with lesion in the pSTS who exhibited difficulty in discriminating facial identity (Fox et al., 2011). Taken together, the double dissociation of cortical region (FFA vs. pSTS) by face task (face memory vs. face perception) reveals the heterogeneous nature of DP’s deficits, as well as a functional division of labor of face-selective regions in the CFN.

In contrast, the overlap analysis with a less stringent threshold suggests a shared neural basis for the deficits of face memory and face perception in the EFN, which likely receives inputs from CFN (Haxby et al., 2000; Axelrod and Yovel, 2012). In fact, the disrupted resting-state function connectivity between the CFN and the EFN is found to account for DPs’ deficits in face processing (Zhao et al., 2018). In this study, we identified the right precuneus and the right OFC in the EFN, whose face selectivity was related to DPs’ performance in both face memory and face perception tasks. Previous studies have found that the precuneus is involved in retrieval of information from episodic memory (Ishai et al., 2000; Burgess et al., 2001) and sensitive to face identity (Kosaka et al., 2003), and the OFC has been hypothesized to guide visual object processing via top-down modulation (Furl, 2015). For example, connectivity between OFC and OFA is modulated by illusory face perception (Li et al., 2010), and connectivity between OFC and FFA was disrupted in DP compared with normal controls (Zhao et al., 2018). Therefore, these EFN regions may engage in both perceptual and mnemonic aspects of face processing, echoing our finding of shared neural correlates of the deficits in face memory and face perception tasks. In other words, although the neural correlates of the deficits in face memory and face perception was separate in CFN, the precuneus and OFC of the EFN demonstrate a joint deficit in both face memory and face perception tasks. Note that the shared neural correlates were found only in a liberal threshold, fitting nicely with previous behavioral studies that some DPs exhibit comorbidity in perceptual and mnemonic deficits, whereas others do not (Behrmann et al., 2005; Duchaine et al., 2007; McKone et al., 2011; Dalrymple et al., 2014; Ulrich et al., 2017).

In summary, our study revealed that both separate and shared neural correlates of face memory and face perception lied in separate face networks, which may help explaining DP’s heterogeneous nature. Besides, our study also provided evidence on the functional division of labor at both cortical and network levels in processing faces. However, there are several questions, which invite future studies to address. First, face perception is traditionally considered as a preceding stage of face memory; that is, the deficit in face perception inevitably leads to the deficit in face memory. However, the weak correlation in behavior and separate neural correlates in the CFN implies that face perception is relatively independent from face memory to some extent. Future studies are needed to illuminate the relation of these two tasks centered in face processing. Second, the comorbidity of the deficits in face memory and face perception was found in the EFN, consistent with the hypothesis that the EFN integrates information from the CFN. However, the connection between CFN and EFN, especially the connection from the FFA and pSTS to the precuneus and frontal regions, is not clear. Future studies with connectivity analyses may illustrate the hierarchy of the face network and provide a more comprehensive neural model on face recognition (Haxby et al., 2000; Ishai, 2008; Zhao et al., 2018). Third, the face perception task used sequential matching paradigm, which likely requires working memory to some extent. Future studies using simultaneous matching paradigm can isolate face perception more completely and examine the relationship between neural correlates underlying face perception and memory in DP. Finally, we did not define the face-selective regions in individual DP participants; future studies using independent localizer runs can define the face regions at individual level and confirm the neural–behavior correlation observed here.

## Data Availability Statement

The raw data supporting the conclusions of this article will be made available by the authors, without undue reservation.

## Ethics Statement

The studies involving human participants were reviewed and approved by the Institutional Review Board of Beijing Normal University. The patients/participants provided their written informed consent to participate in this study.

## Author Contributions

YS and JL designed the study. YS and XTL ran the experiments. XQL analyzed the data. XQL, XTL, YS, and JL wrote the manuscript. All authors contributed to the article and approved the submitted version.

## Conflict of Interest

The authors declare that the research was conducted in the absence of any commercial or financial relationships that could be construed as a potential conflict of interest.

## Footnotes

1 http://www.fmrib.ox.ac.uk/fsl

2 https://afni.nimh.nih.gov/pub/dist/doc/program_help/3dClustSim.html

3 www.brainactivityatlas.org
